# Editorial: The role of retinoic acid signaling in maintenance and regeneration of the CNS: from mechanisms to therapeutic targeting

**DOI:** 10.3389/fnmol.2024.1491745

**Published:** 2024-11-04

**Authors:** Jonathan P. T. Corcoran, Jörg Mey

**Affiliations:** ^1^Neuroscience Drug Discovery Unit, Wolfson Sensory, Pain and Regeneration Centre, King's College London, Guy's Campus, London, United Kingdom; ^2^Hospital Nacional de Parapléjicos, Toledo, Spain; ^3^School of Mental Health and Neuroscience, Maastricht University, Maastricht, Netherlands

**Keywords:** retinoic acid, drug, RAR/RXR, nerve injury, nerve repair, neurodegeneration

There are very few drugs available for neurodegenerative diseases. Due to their complex, multifactorial nature, single-target therapeutic approaches, by far the prevailing modality, are not efficient (de la Fuente et al., 2023). Retinoic acid (RA) signaling is an attractive system to explore in this therapeutic context, as it modulates multiple aspects of the central nervous system (CNS) related to its maintenance and regeneration (Maden, 2007). The Research Topic “*The role of retinoic acid signaling in maintenance and regeneration of the CNS: from mechanisms to therapeutic targeting*” discusses these diverse signaling pathways in various CNS diseases and how drugs can be used to modulate them for therapeutic benefit.

In acute CNS injuries, such as spinal cord injury (SCI), stroke, and traumatic brain injury, RA signaling promotes tissue repair (Goncalves et al., 2015; Hummel et al., 2020; Kang et al., 2023). RA also modulates various pathways involved in the pathophysiology of neurodegenerative diseases, namely Alzheimer's disease (AD; Shudo et al., 2009), Parkinson's disease (PD; Marie et al., 2021), Huntington's disease (Niewiadomska-Cimicka et al., 2017), and motor neuron disease (Corcoran et al., 2002). RA signaling can also modulate the immune system, inflammation, and phagocytosis, which are thought to play a critical role in the development and progression of these diseases (Behl et al., 2022; Wu et al., 2021).

The RA signaling pathway acts via the nuclear receptors, the RA receptors (RARs) and the retinoid X receptors (RXRs), of which there are three types each and various subtypes (Maden, 2007). For signaling to occur a RAR/RXR heterodimer forms that binds to retinoic acid response elements (RARES) upstream of the target genes, RA binds and transcription occurs (Huang et al., 2014). It is thought that RA can regulate up to 20% of the genome (Luo et al., 2009; Mey, 2017). It is this regulation of numerous pathways that makes targeting the RARS/RXRs an attractive drug option to treat CNS diseases. The RXRs can also bind to nuclear orphan receptors, which further adds to the complexity of signaling (Sharma et al., 2022).

While originally regarded as nuclear signaling, it is now recognized that RA can exert important functions through non-canonical pathways. Piazza et al., “*Non-canonical retinoid signaling in neural development, regeneration and synaptic function*” review these in detail and discuss their importance in activating protein kinase C (PKC), PI3K/AKT3, and ERK1/2 signaling in neural development, nerve regeneration, and synapse formation. As these pathways are highly relevant clinically, synthetic retinoids have been developed that induce axonal regeneration by activating genomic (RAR/RXR) and non-genomic (ERK1/2 kinase) pathways (Khatib et al., 2019).

Despite the multitude of regenerative pathways governed by RA signaling, all-*trans* RA itself is not an attractive CNS therapeutic. Not only does it have poor drug-like properties, is associated with liver toxicity, and is rapidly catabolized by CYP26 enzymes, but it is also a pan-RAR agonist. Since these receptors are widely expressed, undesirable effects, both inside and outside the CNS, are likely to occur at pharmacological doses. In addition, the spatiotemporal fine-tuning of activation of specific RARs in repair mechanisms, such as in spinal cord regeneration for instance, where coordination of RARα and β is required, cannot be achieved with RA (Goncalves et al., 2019b). The challenge is to synthesize receptor-specific drugs that overcome current toxicities (Borthwick et al., 2020) and/or to target retinoids to the area of interest, using for example liposomes (Ferreira et al., 2020).

To date there are only six retinoids that have entered the clinic, these are pan-RARs, tretinoin, a gel formulation; isotretinoin, and acetretin, oral formulations (Baldwin et al., 2023; Layton, 2009; Khalil et al., 2017); a RARβ/γ agonist, adapalene, a gel formulation for the treatment of acne (Rusu et al., 2020); one is a RARβ/α agonist, tamibarotene, for the treatment of acute promyelocytic leukemia (Nagai and Ambinder, 2023); and an RXR agonist, bexarotene, for cutaneous T-cell lymphoma (Scarisbrick et al., 2013).

Recent work has developed a first-in-class RARβ selective agonist, C286, that presents unique therapeutic advanatages as it is less lipophilic than current clinical retinoids, evading retinoids typical hepatic toxicity, has good oral availability and can cross the blood-brain barrier (Goncalves et al., 2019a).

The contribution by Goncalves et al., “*C286, an orally available retinoic acid receptor* β *agonist drug, regulates multiple pathways to achieve spinal cord injury repair*” further validates the use of C286 to stimulate axonal/neurite outgrowth and highlights the numerous pathways it regulates to achieve this effect. These pathways are involved in synaptogenesis, axonal outgrowth and modulation of the extracellular matrix, and are present in different rodent models of nerve injury, and across species, human-derived neurons significantly increased neurite outgrowth in response to C286 treatment. This is important as it suggests that RARβ signaling by C286 evokes the same reparative mechanisms in different nerve injuries and different species, thus being predictive of successful efficacy in humans. The dose used in the proof-of-concept (POC) studies does not exceed the no-observed-adverse-effect level (NOAEL), and the use of clinical retinoids that are not RARβ selective does not result in functional outputs in nerve injury. Taken together the data suggest that phase IIA trials are warranted in acute nerve injury and given the cross-over in pathways with chronic CNS injury the drug may be of use here as well.

With regards to RXR signaling, given the difficulty in developing specific agonists with a good safety profile, one possible approach is to target the orphan nuclear receptor they partner with. For instance, Deckmyn et al., “*Farnesoid X receptor activation in brain alters brown adipose tissue function via the sympathetic system*” show that the farnesoid X receptor (FXR) is expressed in the hypothalamus and is involved in energy homeostasis. They activate the FXR/RXR with the FXR agonist GW4064, thus negating the use of an RXR agonist such as bexarotene and achieving a more specific outcome. The delivery of the agonist is done by intracerebroventricular (ICV) treatment to avoid activation of peripheral FXR. Similarly, Zhang et al., “*Regulation of nuclear factor erythroid-2-related factor 2 as a potential therapeutic target in intracerebral hemorrhage*” review the role of Nuclear factor erythroid-2-related factor 2 (Nrf2) in preventing oxidative stress in stroke and therefore mitigating brain injury. There are a number of pathways discussed that can increase the expression of NrF2, and one of these is the nuclear orphan receptor, Peroxisome proliferator-activated receptor γ (PPARγ). Agonists of PPARγ include thiazolidinediones, which are used to treat diabetes mellitus type II, although like current FXR agonists they need to be developed for better brain penetration to treat CNS diseases and/or a better delivery mechanism.

In conclusion, the Research Topic has uncovered new pathways involved in CNS repair that are under the regulation of retinoid signaling. This provides a fertile platform for further target validation and translational research studies to pave the way for novel therapeutic developments for a wide umbrella of neurodegenerative diseases.
